# Effect of Interventions on Learning Burnout: A Systematic Review and Meta-Analysis

**DOI:** 10.3389/fpsyg.2021.645662

**Published:** 2021-02-26

**Authors:** Lei Tang, Fan Zhang, Ruoyun Yin, Zhaoya Fan

**Affiliations:** School of Public Health and Management, Research Center for Medicine and Social Development, Collaborative Innovation Center of Social Risks Governance in Health, Chongqing Medical University, Chongqing, China

**Keywords:** learning burnout, interventions, students, meta-analysis, effectiveness

## Abstract

**Objectives:** This study aimed to provide a comprehensive understanding of all intervention for learning burnout by meta-analyzing their effects.

**Methods:** Relevant studies that had been published up to September 18, 2020, were identified through a systematic search of the PubMed, Web of Science, the China National Knowledge Infrastructure (CNKI), and Wan Fang databases. Eligible studies included randomized control trials of any learning burnout intervention conducted among students. The Jadad scale was used to evaluate the quality of the study. Random-effect meta-analyses, subgroup analyses, meta-regression analysis, and sensitivity analysis were conducted. Funnel plots and Egger's tests were used to evaluate publication bias. Duval and Tweedie's non-parametric trim-and-fill method was used to adjust the effect of publication bias.

**Results:** Of the 5,245 articles found, 39 met the inclusion criteria for the systematic review. There were 3,400 students, including 1,847 students in the intervention group and 1,553 in the control group. A meta-analysis of 44 studies showed that the interventions were effective. Subgroup analyses were conducted according to education, scales, intervention measures, and intervention time. The results showed that, compared with the control group, the learning burnout scores of undergraduates, secondary vocational students, and middle school students were significantly lower. Based on different scales, all interventions were also effective. The funnel plot was asymmetric and consistent with the results of Egger's test. The trim-and-fill method was used, and seven missing studies were merged to obtain a symmetric funnel plot.

**Conclusions:** This meta-analysis indicated that learning burnout interventions are effective. The subgroup analyses showed that group counseling is the most widely used, exercise intervention is probably the most effective, and 8 weeks or more is the appropriate intervention time. An integrated intervention study based on the factors of learning burnout adds value. More studies are needed to supplement the results in the future.

## Introduction

Burnout was first proposed by Freudenberger (1974) and used to describe a state of exhaustion caused by excessive working hours and intensity among social workers, including medical personnel. Later, the concept of learning burnout was coined by Kurzman (1981) to describe students' negative attitudes toward their studies and school activities as well as their depleted mental state. Among other researchers, including Meier and Schmeck (1985), Neumann et al. (1990), Maslach and Leiter (1997), Slivar (2001), Lian et al. (2005), and Hu and Dai (2006), the most popular view is that of emotional exhaustion, academic alienation, and a low sense of achievement caused by students' excessive learning needs (Schaufeli et al., 2002).

Learning burnout reflects students' negative psychology and affects their mental health, academic performance, and interpersonal relationships (Lian et al., 2005). Brought on by long-term pressure and burdens, it is easy for students to experience burnout (Chou et al., 2014; Abarghouei et al., 2016). A cross-sectional study of 542 primary school students found high burnout among 33.6% of sixth graders and 27.5% of fourth—and fifth-graders (Tavolacci and Veber, 2015). In addition, the prevalence of burnout among 662 undergraduates was also found to be 7.4%, and burnout symptoms had a significant predictive effect on depression (Al-Alawi et al., 2017). A cross-sectional study of incoming medical students also found high levels of burnout (Santos et al., 2018). They exhaust their energy and gradually lose their enthusiasm for learning, and the psychological problems start impacting their physical health. Sometimes learning burnout may cause physical diseases (including hypertension, arteriosclerosis) (May et al., 2015b), negative psychological and behavioral problems (such as depression, truancy, and dropout) (May et al., 2015a), even substance abuse and the increased possibility of suicidal thoughts among students (Dyrbye et al., 2008; Jackson et al., 2016).

Some scholars have explored interventions that may improve students' learning burnout. Using a social-cognitive approach (Bresó et al., 2010), successfully reduced burnout levels with interventions based on self-efficacy. Skodova and Lajciakova (2013) used psychosocial training to intervene the burnout of students majoring in health care, and concluded that psychosocial training could be considered as an effective tool to prevent burnout of professional helpers. Zhu (2016) interfered with junior high school students through box room interventions, and Shi (2017) interfered with sixth grade students through recruiting subjects and carrying out class group counseling, both of which achieved good results. Khalaj and Savoji (2018) used cognitive self-regulation strategies to reduce the students' academic burnout. Although these interventions vary—burnout's manifestations and causes differ—most aim to help individuals express negative emotions and eliminate behaviors that do not promote individuation (Chen et al., 2012). Most commonly, interventions include various sports, box room interventions, and group or psychological counseling. Furthermore, the level of burnout can be reduced by improving an individual's coping abilities (including cognitive stress management, relaxation training, time management, or social training) (Pines, 2000) and using team self-management interventions (Elloy et al., 2001).

This study aims to provide a comprehensive understanding of all the interventions on learning burnout, by meta-analyzing their effects. We hope to understand the intervention status and various scales of the measure of learning burnout, learning burnout interventions and its details, and the effect of common interventions, hope to provide evidence and ideas for further studies on learning burnout.

## Methods

This systematic review was registered with the International Prospective Register of Systematic Reviews (PROSPERO), number CRD42020214846, and conducted according to the Preferred Reporting Items for Systematic Reviews and Meta-Analyses (PRISMA) statement (Moher et al., 2009).

### Study Strategy

We analyzed all the related studies that had been published up to September 18, 2020. Using the terms “learning burnout” or “academic burnout” or “student burnout” or “school burnout,” title/abstract retrieval was adopted in PubMed and Web of Science and in the China National Knowledge Infrastructure (CNKI), and Wan Fang, title/keyword/abstract retrieval was used. References of the articles that we found were also searched and tracked for potentially missed articles.

### Selection Criteria

Based on a review of the retrieved article titles, abstracts, and full texts, studies were selected if they met the following inclusion criteria: (1) randomized controlled trials, (2) interventions focused on learning burnout, (3) published in English or Chinese, (4) the most recent among multiple publications of the same study. We excluded: (1) articles with duplicate, incomplete, or inaccessible data (not obtainable from the original author), (2) self-controlled trials, reviews, news reports, meeting minutes, commentaries, editorials, and protocol. Therefore, the studies included were not limited to the population. Randomized controlled studies with complete data using any intervention that can affect learning burnout and setting blank or placebo control groups can be accepted.

### Data Extraction

Two authors (TL and YRY) independently screened all the literature and extracted data, while cross-checking the results. If there were differences in the screening or data extraction processes between the two authors, they discussed the matter, and a third author (ZF) may also get involved. Strictly adhering to the inclusion criteria guidelines, we first screened the titles and abstracts. Then, the full text was obtained and secondary screening was conducted for the reserved and questionable articles from the primary screening. For each study included in the systematic review, the following data from the intervention and control groups were extracted: first author, year of publication, study location, educational level of the participants, scales used, intervention measures, intervention frequency, intervention times, number of samples, mean and standard deviations, or the standard deviation of the mean difference and the mean deviation of the pre- and post-intervention.

### Assessment of Study Quality

Two authors (TL and FZY) independently conducted the quality evaluation of the studies using the Jadad scale. It consists of three domains: methods to generate randomization sequences (0–2 points), double blinding (0–2 points), and withdrawal and dropouts (0–1 points) (Jadad et al., 1996). The quality of the studies was classified as high (3–5 points) or low (0–2 points). The authors' results were compared, and a third author (ZF) intervened if consensus could not be reached.

### Statistical Analysis

STATA software (version 15) was used for the meta-analysis. The scale score of learning burnout is a continuous variable. Some studies only provided the score of each dimension rather than the total score of the whole scale. The means and standard deviation of the total score were calculated using the Cochrane Handbook for Systematic Reviews of Interventions (Cumpston et al., 2019). The standardized mean difference (SMD) and a 95% confidence interval (95% CI) were used in this study because of the different measurement tools of the included studies. For any test or model, we considered *p* < 0.05 as statistically significant.

We used Cochran's χ^2^-based *Q*-test and the *I*^2^-test to assess the heterogeneity of the studies (Higgins and Thompson, 2002). For those with statistical heterogeneity (defined as *p* ≤ 0.10 or *I*^2^ ≥ 50%) the random effects model of DerSimonian and Laird was used, and the fixed effects model of Mantel-Haenszel for meta-analysis, for studies without significant heterogeneity (defined as *p* > 0.10 or *I*^2^ <50%) (Overton, 1998). To solve the heterogeneity among studies, we analyzed the educational background, scales, and intervention measures and time, through subgroup analyses. Where necessary, a meta-regression analysis was conducted, and to ensure stability, individual studies that may affect the results were excluded through a sensitivity analysis. We evaluated and tested publication bias with Funnel plots and Egger's tests (Egger et al., 1997; Wang et al., 2008) and used Duval and Tweedie's non-parametric trim-and-fill method to adjust the effect of the bias (Peters et al., 2007).

## Results

### Search Results

**Figure 1:**
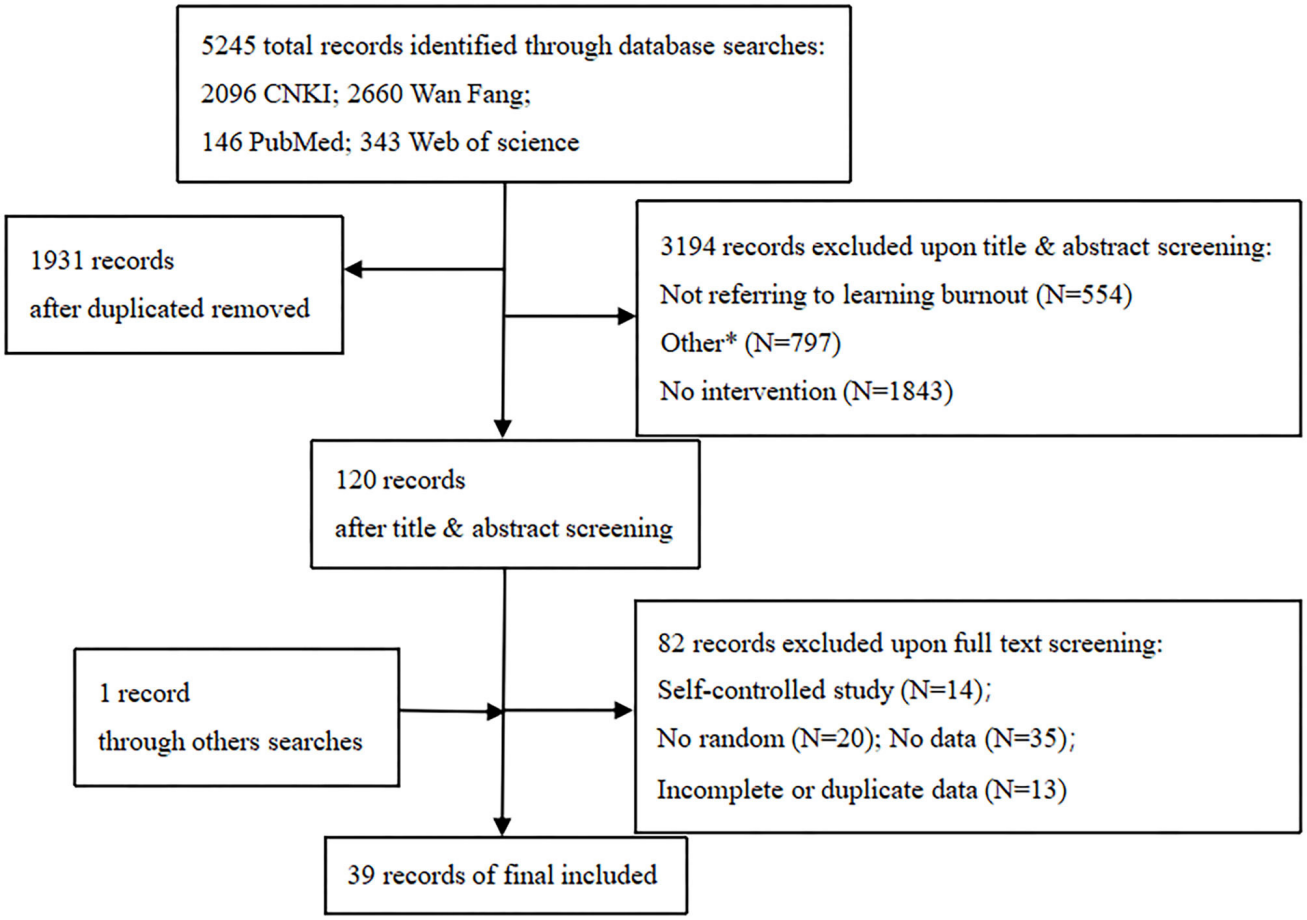
Flow Chart of Study Selection. Flow chart shows literature search for relevant studies about interventions on learning burnout. *Studies were excluded because they were either news reports, commentaries, editorials or a review, protocol.

Of the 5,245 citations initially identified, 1,931 were excluded due to repetition and 3,194 following further review of titles and abstracts. After the full-text screening of 120 studies, 34 were excluded because they were not randomized controlled trials and 48 because they contained no, incomplete, or duplicate data. After reviewing the references of the remaining studies (Ni and Wu, 2009), was found to meet the inclusion criteria and was added to our selection. Finally, a total of 39 studies involving 3,400 students were included. The screening process is shown in Figure 1.

### Characteristics of Included Studies

The 39 articles were published as follows: 2009 (*n* = 1) (Ni and Wu, 2009), 2011 (*n* = 1) (Zhou, 2011), 2012 (*n* = 4) (Chen et al., 2012; Luo X., 2012; Luo Z. Y., 2012; Wang, 2012), 2013 (*n* = 2) (Liu, 2013; Xiao, 2013), 2014 (*n* = 4) (Dai, 2014; Xia and Yang, 2014; Yang, 2014; Zhang et al., 2014), 2015 (*n* = 2) (Yao, 2015; Zhang, 2015), 2016 (*n* = 7) (Guo, 2016; Li, 2016; Shang, 2016; Sheng, 2016; Tan, 2016; Wang, 2016; Wang et al., 2016), 2017 (*n* = 5) (Wu X. Q., 2017; Wu Y. P., 2017; Xiong and Fang, 2017; Ye et al., 2017; Zhang, 2017), 2018 (*n* = 4) (Huang, 2018; Wu, 2018; Xu, 2018; Zhang, 2018), 2019 (*n* = 6) (Ezenwaji et al., 2019; Li, 2019; Xi, 2019; Xu, 2019; Zhao, 2019; Zhou, 2019), and 2020 (*n* = 3) (Chen, 2020; Shen, 2020; Wan, 2020). Of the 39 articles 38 were published in Chinese and one in English. Of the 3,400 students, 1,847 were in the intervention group and 1,553 in the control group.

Among the Chinese articles, 11 did not include the specific research area. The remaining 27 were conducted in the following Chinese provinces: one each in Heilongjiang (Ni and Wu, 2009), Tianjin (Wang, 2012), Yunnan (Yang, 2014), Beijing (Zhang et al., 2014), Gansu (Zhang, 2015), Shanxi (Guo, 2016), Shanghai (Xu, 2018), Liaoning (Huang, 2018), and Guangdong (Chen, 2020); two each in Jiangsu (Sheng, 2016; Zhang, 2018) and Jiangxi (Wang, 2016; Xiong and Fang, 2017); three each in Henan (Luo Z. Y., 2012; Yao, 2015; Xi, 2019) and Hebei (Xia and Yang, 2014; Tan, 2016; Zhao, 2019); and four each in Zhejiang (Dai, 2014; Shang, 2016; Wu X. Q., 2017; Wu, 2018) and Fujian (Chen et al., 2012; Wu Y. P., 2017; Ye et al., 2017; Xu, 2019). The research conducted beyond China was conducted in Nigeria, West Africa (Ezenwaji et al., 2019). The research covered primary and middle schools, and universities. There were articles for postgraduates (*n* = 2), undergraduates (*n* = 13), junior college students (*n* = 3), secondary vocational students (*n* = 5), middle school students (*n* = 14), and primary school students (*n* = 2).

Among the 15 scale types to measure learning burnout, the top three were: Learning Burnout Scale of College Students (LBSCS) of Lian et al. (2005), the Adolescent Student Burnout Inventory (ASBI) of Wu et al. (2010), and the Middle School Students Learning Burnout Scale (MSSLBS) of Hu and Dai (2006). The English paper used the Oldenburg Burnout Inventory Student Version (OLBI-S) (Bonini et al., 2011). In Chinese studies, the most common interventions were group counseling, then sports intervention, time management training, box room intervention, and so on. The English article included rational emotive behavior coaching (REBC). The intervention time in the articles (except for four that did not mention it) ranged from two to 18 weeks.

The results of the Jadad scale were as follows: the English article scored five points (high-quality); one Chinese study scored four points and six Chinese studies scored three points (all high-quality). The other 31 articles were of low quality. The detailed characteristics of the included studies are shown in Table 1.

**Table 1:** Characteristics of included studies.

**References**	**Location**	**Population**	**Scale**	**Times**	**Frequency**	**Simple**	**Interventions**	**Control**	**Jadad**
Ni and Wu (2009)	Heilongjiang	Undergraduate	I	4 weeks	The retest was performed after 5 weeks	10/19	Cognitive-behavioral group counseling	No	2
Zhou (2011)	–	Undergraduate	II	2 mouths	3 times a week, Moderate intensity exercise for 1 h each time	32/17 34/18	Antagonistic sports Non-antagonistic sports	No	3
Chen et al. (2012)	Fujian	Undergraduate	II	8 weeks	Once a week, about 2 h each time	30/30	Group box room intervention	No	3
Luo X. (2012)	–	Postgraduate	X III	4weeks	Twice a week, 8 times in total	15/15	Group psychological counseling	No	2
Luo Z. Y. (2012)	Henan	Middle school student	III	8 weeks	Once a week, about 40 min each time	70/68	Time management training	No	2
Wang (2012)	Tianjin	Undergraduate	VI	16 weeks	Once a week, 2 class h each time	150/150	Specific teaching instruments	No	2
Liu (2013)	–	Undergraduate	VII	–	Once a week, 2–2.5 h each time	1010/	Combined psychoeducation, group and individual counseling	No	2
Xiao (2013)	–	Postgraduate	VIII	8 weeks	Once a week, about 90 min each time	15/15	Group psychological counseling	No	2
Dai (2014)	Zhejiang	Middle school student	IV	8 weeks	Once a week, about 90 min each time	24/24	Study inputs into training	No	2
Xia and Yang (2014)	Hebei	Schoolchild	III	7 weeks	Once a week	41/33	Mental health education	No	2
Yang (2014)	Yunnan	Secondary vocational student	II	7 weeks	–	62/90	Enterprise no boundary class management	Routine institutional management	2
Zhang et al. (2014)	Beijing	Undergraduate	II	7 weeks	Once a week, 3 h each time, and retested after 1 month	10/10	Group counseling	No	2
Yao (2015)	Henan	Middle school student	IV	8 weeks	Once a week, about 90 min each time	25/25	Group psychological counseling	No	2
Zhang (2015)	Gansu	Middle school student	III	5 weeks	Once a week, about 1 h each time	11/12	Mindfulness based stress reduction therapy	No	2
Guo (2016)	Shanxi	Undergraduate	II	1 mouth	8 times in total, and retest after 3 months	35/35	Group counseling	No	2
Li (2016)	–	Middle school student	X IV	8 weeks	Twice a week, 50 min each time	30/30	Emotional teaching	Routine teaching	3
Sheng (2016)	Jiangsu	Middle school student	X V	18 weeks	Three times a week, 45 min each time	72/72 74/74 79/79	Exercises intervention	No	2
Tan (2016)	Hebei	Middle school student	IX	3 mouths	Once a week, 45 min each time	30/30	An intervention program based on attribution theory, interpersonal interaction theory, and the theory of burnout and job matching model	No	2
Wang (2016)	–	Middle school student	IV	1 mouth	–	143/49 154/52 143/49	Only received group psychological counseling Only did mental health gymnastics Two intervention combinations	No	2
Wang (2016)	Jiangxi	Secondary vocational student	III	8 weeks	Once a week, 1.5–2 h each time, and retest after 4 months	21/21	Time management training	No	2
Shang (2016)	Zhejiang	Junior college student	II	6 weeks	Once a week, 1.5 h each time	15/15	Solution-focused group counseling	No	2
Wu Y. P. (2017)	Fujian	Secondary vocational student	XII	3 weeks	Once a week, 20 min each time	30/30	Standard imaginary contact	Landscape imagination contact	2
Xiong and Fang (2017)	Jiangxi	Undergraduate	II	–	A total of 7	41/44	Cognitive-behavioral group counseling	Simple activities	2
Ye et al. (2017)	Fujian	Undergraduate	II	2 weeks	Once a week, 30 min each time	30/30	Group mindfulness intervention	No	2
Zhang (2017)	–	Middle school student	III	6 weeks	Once a week, 2 h each time	49/50	Group psychological counseling	No	3
Wu X. Q. (2017)	Zhejiang	Junior college student	X	8 weeks	Once a week, 1.5–2 h each time	30/30	Group counseling	Mental health course	2
Wu (2018)	Zhejiang	Middle school student	V	–	From 2016.11 to 2017.06	41/41	Prompt review training	General teaching	3
Xu (2018)	Shanghai	Middle school student	IV	8 weeks	Once a week, 50 min each time	9/9	Group counseling	No	2
Zhang (2018)	Jiangsu	Secondary vocational student	III	8 weeks	Once a week, 90 min each time	12/12	Group psychological counseling	No	2
Huang (2018)	Liaoning	Middle school student	III	6 weeks	Once a week	37/32	Group counseling	No	2
Ezenwaji et al. (2019)	Nigeria	Undergraduate	XI	12 weeks	Twice a week, follow-up which was conducted 3 months from the end of the group coaching	26/26	Rational emotive behavior coaching, REBC	No	5
Li (2019)	–	Middle school student	IV	8 weeks	Once a week, 90 min each time	39/39	Group counseling	Routine psychological course	2
Xi (2019)	Henan	Junior college student	II	–	–	32/32	General interventions	Normal teaching	3
Xu (2019)	Fujian	Undergraduate	II	8 weeks	Once a week, 2 h each time	15/15	Vocational literacy orientation group mentoring	No	2
Zhao (2019)	Hebei	Schoolchild	III	8 weeks	Once a week, 40 min each time	30/30	Psychological resilience group counseling	No	2
Zhou (2019)	–	Middle school student	IV	8 weeks	Six days a week, Once a day, 10–15 min each time	16/16	Mindfulness meditation interventions	Listen to biographies of people from the Forum	2
Chen (2020)	Guangdong	Secondary vocational student	IV	8 weeks	Once a week, 2 h each time	25/25	Group counseling	No	2
Shen (2020)	–	Undergraduate	II	8 weeks	Once a week, 2 h each time, and retest after 3 months	20/20	Group counseling	No	2
Wan (2020)	–	Undergraduate	II	2 weeks	Once a week, 30 min each time	30/30	Group mindfulness intervention	No	4

*This table was sorted by year of publication. I is a modified Maslach Burnout Inventory-Student (MBI-S); II is the Learning Burnout Scale of College Students (LBSCS) of Lian Rong; III is the Adolescent Student Burnout Inventory (ASBI) of Wu Yan; IV is the middle school students learning burnout scale (MSSLBS) of Hu Qiao; V is a four-point scale of the middle school students learning burnout scale of Hu Qiao; VI is the learning burnout scale of college students of Chen Yan; VII is a seven-point scale of the learning burnout scale of college students of Li Yongxin; VIII is the master's postgraduate learning burnout questionnaire of Li Nana; IX is the middle school students learning burnout scale of Xing Qiang; X is the learning burnout questionnaire of college students of Cao Jintyuan; XI is Oldenburg Burnout Inventory Student Version (OLBI-S); XII is modified LBSCS; XIII is homemade master's postgraduate learning burnout questionnaire; XIV is self-made history course learning burnout questionnaire for high school students; XV is a 10-point scale of self-made learning burnout questionnaire for high school female students*.

### Interventions of Learning Burnout

#### Overall Analysis

**Figure 2:**
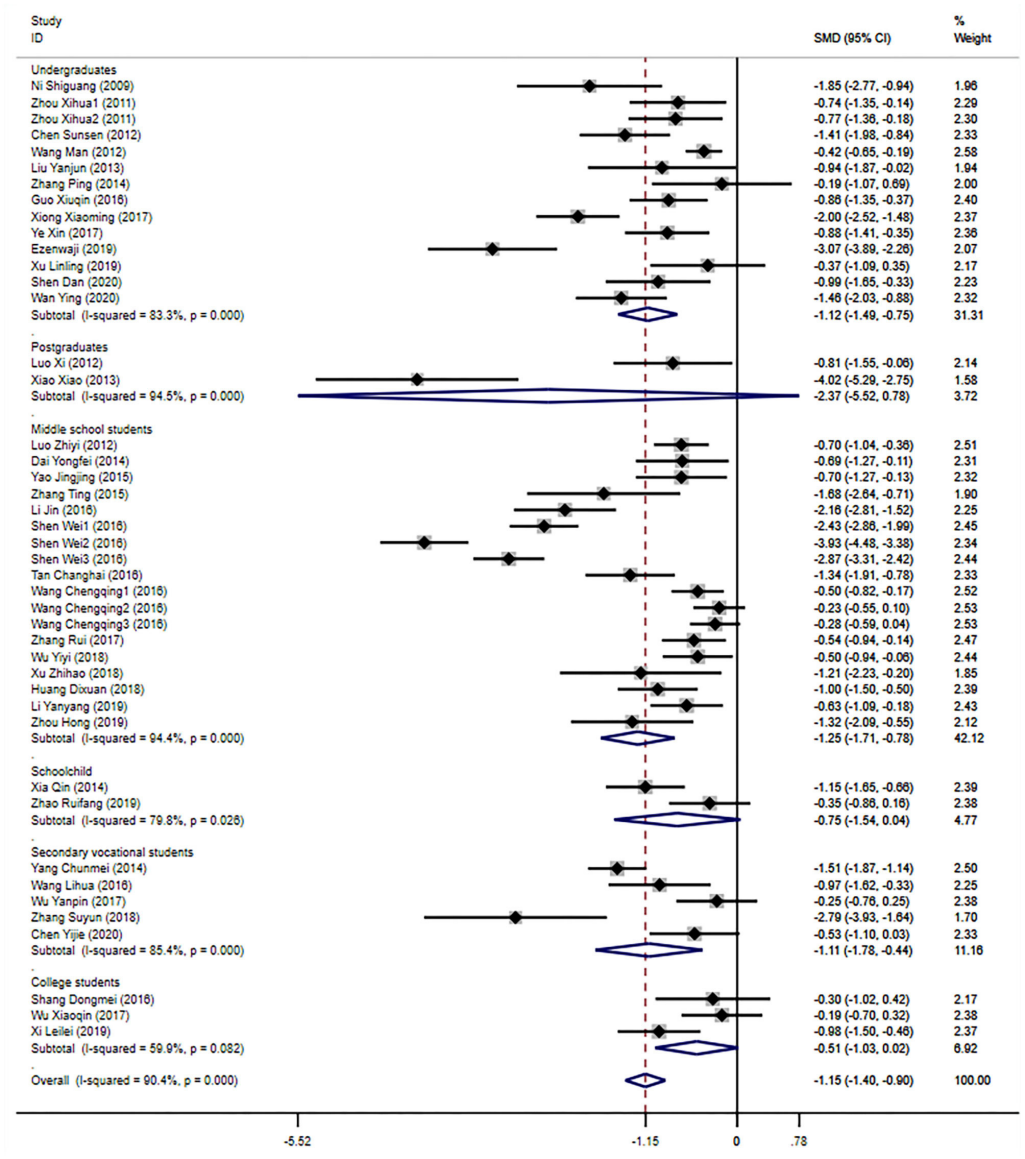
Overall analysis and subgroup analysis based on educational background.

Compared with the control group, the scores of learning burnout were significantly decreased under the random effects model (SMD −1.15, 95% CI: −1.40, −0.90, *p* < 0.001, *I*^2^ = 90.4%), shown in Figure 2. Sheng (2016) analysis three grades of senior high school, so it is considered as three studies in the meta-analysis. Zhou (2011) involved two exercise interventions, which randomly divided the subjects into three groups (2 = experimental, 1 = control), referring to the Cochrane Handbook. The control groups were all divided into two groups, so that their mean and standard deviation were unchanged, regarded as two studies. Similarly, Wang et al. (2016) randomized the included subjects into four groups (3 = experimental, 1 = control) and divided the number of control subjects equally into three groups—the mean and standard deviations of which remained the same. After performing the analysis, three studies were considered. Therefore, the overall analysis included 44 studies.

#### Subgroup Analyses

Subgroup analyses were conducted according to education, scales, intervention measures, and intervention time.

The subgroup analysis according to the participants' educational backgrounds showed that, compared with the control group, the learning burnout scores of undergraduates, secondary vocational students, and middle school students were significantly lower separately [undergraduate: SMD −1.12, 95% CI: [−1.49, −0.75], *p* < 0.001, *I*^2^ = 83.3%; secondary vocational students: SMD −1.11, 95% CI: [−1.78, −0.44], *p* < 0.001, *I*^2^ = 85.4%; middle school students: SMD −1.25, 95% CI: [−1.71, −0.78], *p* < 0.001, *I*^2^ = 94.4%]. However, in the analyses of postgraduates, junior college students, and schoolchildren, no significant effect was found in the intervention group compared with the control group [postgraduates: SMD −2.37, 95% CI: [−5.52, 0.78], *p* < 0.001, *I*^2^ = 94.5%; Junior college students: SMD −0.51, 95% CI: −1.03, 0.02], *p* = 0.082, *I*^2^ = 59.9%; schoolchildren: SMD −0.75, 95% CI: [−1.54, 0.04], *p* = 0.026, *I*^2^ = 79.8%].

The subgroup analysis of the learning burnout scale showed that the effect of the intervention group was significantly lower than that of the control group. Compared with the control group, the exercise intervention group had significantly lower learning burnout, and the intervention effect was significantly higher than group counseling, time management training, and other intervention measures [group counseling: SMD −0.93, 95% CI: [−1.22, −0.65], *p* < 0.001, *I*^2^ = 79.3%; exercise intervention: SMD −2.16, 95% CI: [−3.26, −1.06], *p* < 0.001, *I*^2^ = 95.6%; time management training: SMD −0.76, 95% CI: [−1.07, −0.46], *p* = 0.461; others: SMD −1.08, 95% CI: [−1.40, −0.76], p < 0.001, *I*^2^ = 86.2%].

Subgroup analysis of intervention time showed that different intervention times had a significant intervention effect. The intervention group with the intervention period ≥8 weeks has the best intervention effect on learning burnout [≤ 4 weeks: SMD −0.70, 95% CI: [−1.00, – 0.40], *p* < 0.001, *I*^2^ = 72.5%; 4–8 weeks: SMD −0.93, 95% CI: [−1.33, −0.53], *p* = 0.002, *I*^2^ = 72.0%; ≥8 weeks: SMD −1.40, 95% CI: [−1.82, −0.98], *p* < 0.001, *I*^2^ = 93.2%; not explained: SMD −1.11, 95% CI: [−1.82, −0.40], *p* < 0.001, *I*^2^ = 84.1%], as shown in Table 2.

**Table 2:** Subgroup analysis of the included studies.

	***N***	**SMD (95%CI)**	***P***	***I*^**2**^**	**Publication bias**				**Effect model**
					***Z***	**P_**Begg**_**	**t**	**P_**Egger**_**	
**Scale**									
LBSCS	13	−1.01 (−1.29 to −0.74)	<0.001	66.6%	2.44	0.015	5.25	0.013	Random
ASBI	8	−0.97 (−1.32 to −0.63)	0.036	68.7%	−1.48	0.138	−4.03	0.020	Random
MSSLBS	10	−0.52 (−0.69 to −0.34)	0.210	25.4%	−3.13	0.002	−3.00	0.001	
Others	13	−1.84 (−2.58 to −1.10)	<0.001	96.0%	−0.61	0.542	−5.35	0.095	Random
**Interventions**									
Group counseling	21	−0.93 (−1.22 to −0.65)	<0.001	79.3%	−2.17	0.030	−3.91	0.008	Random
Exercise intervention	5	−2.16 (−3.26 to −1.06)	<0.001	95.6%	0.49	0.624	14.43	0.403	Random
Time management training	2	−0.76 (−1.07 to −0.46)	0.461		−1.00	0.317	−1.80		Random
Others	16	−1.08 (−1.40 to −0.76)	<0.001	86.2%	−2.43	0.015	−4.40	0.006	Random
**Time**									
≤ 4 weeks	9	−0.70 (−1.00 to −0.40)	<0.001	72.5%	−2.09	0.037	−4.45	0.011	Random
4–8 weeks	7	−0.93 (−1.33 to −0.53)	0.002	72.0%	0.15	0.881	1.63	0.520	Random
≥8 weeks	24	−1.40 (−1.82 to −0.98)	<0.001	93.2%	−2.13	0.033	−3.99	0.059	Random
Not explained	4	−1.11 (−1.82 to −0.40)	<0.001	84.1%	−1.36	0.174	−1.64	0.820	Random

*LBSCS is Learning Burnout Scale of College Students compiled by Lian Rong; ASBI is the Adolescent Student Burnout Inventory compiled by Wu Yan; MSSLBS is the middle school students learning burnout scale compiled by Hu Qiao*.

#### Meta-Regression Analysis and Sensitivity Analysis

To identify the possible sources of heterogeneity, the meta-regression model was used to calculate the factors related to heterogeneity, including educational level, scales, intervention measures, and intervention time. The results showed that these variables were not statistically significant. Furthermore, we used the sensitivity analysis of the random effects model to evaluate the impact of each study on the combined results and identify potential sources of heterogeneity. The results did not show strong evidence of the impact of individual studies on the overall results.

### Publication Bias

**Figure 3:**
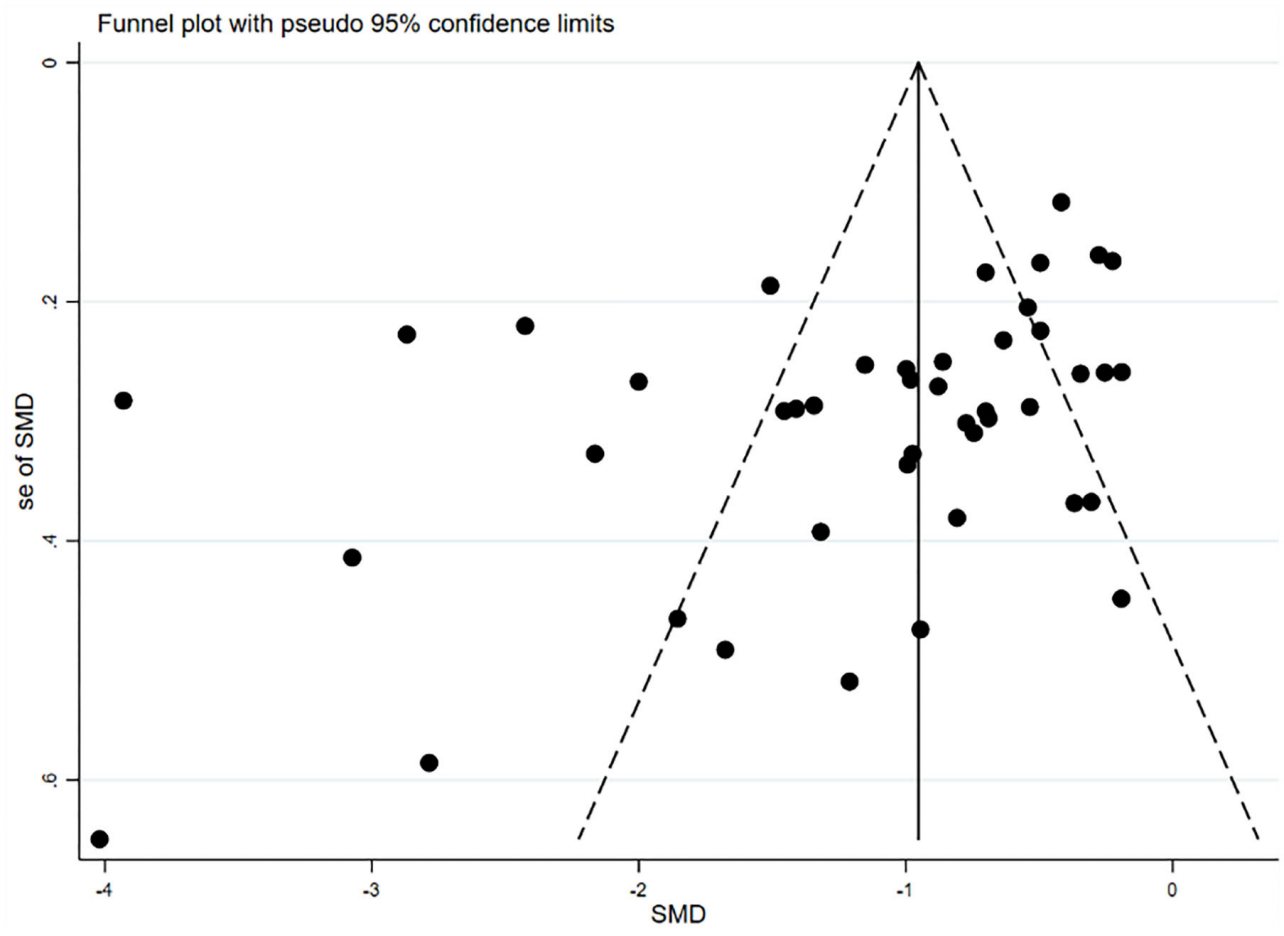
Funnel plot of interventions of learning burnout.

**Figure 4:**
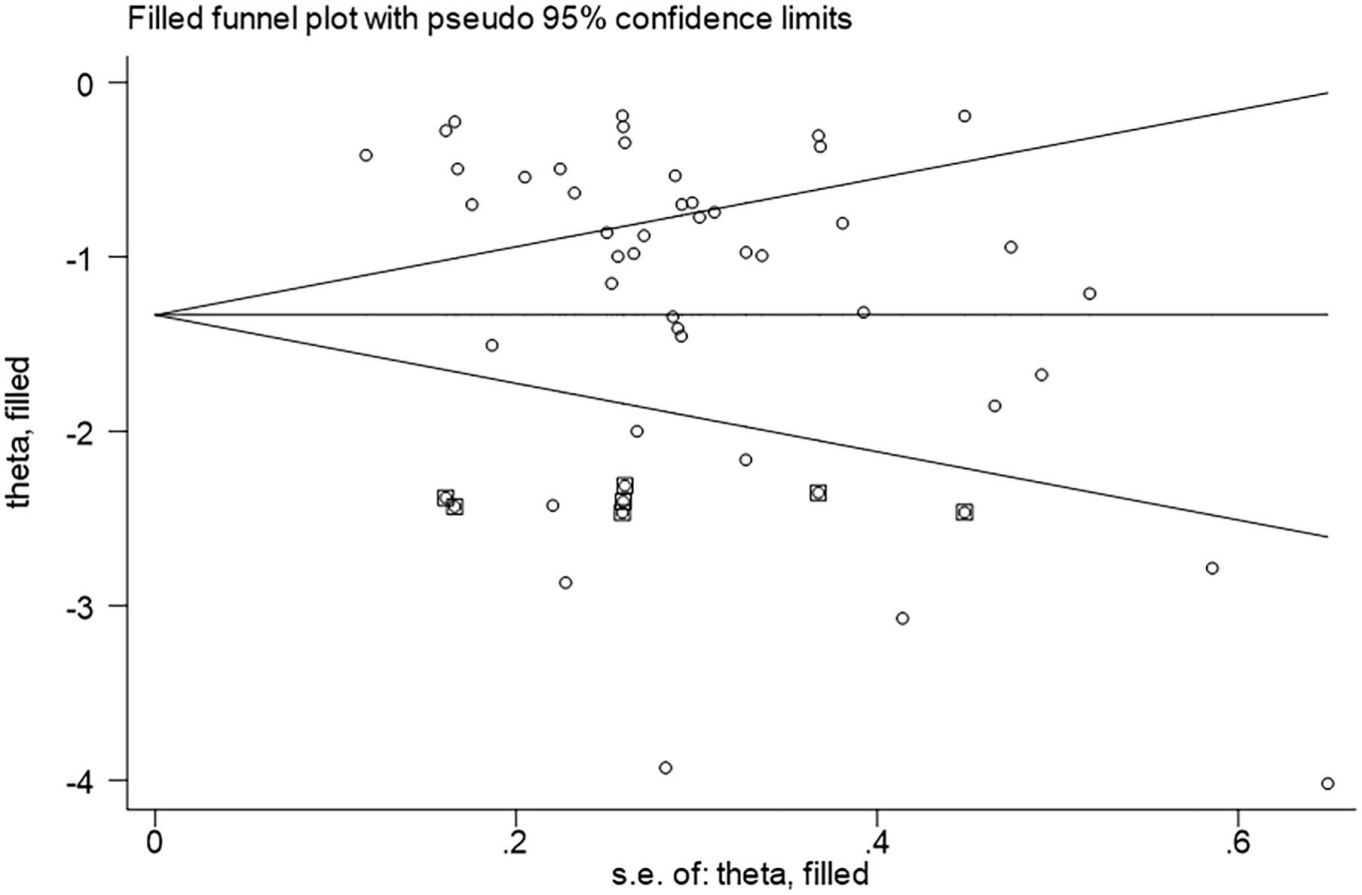
Trim and fill method for evaluating publication bias (the circles alone are real studies and the circles enclosed in boxes are “filled” studies. The horizontal line represents the summary effect estimates, and the diagonal lines represent pseudo-95% CI limits).

A funnel plot was produced to determine whether significant publication bias was present. Relatively symmetrical figures were interpreted as a lack of significant publication bias. The results showed that the funnel plot was asymmetric (Figure 3), consistent with the results of Egger's linear regression test (*p* = 0.01). Duval and Tweedie's non-parametric trim-and-fill method was used to adjust the effect of publication bias since it can make up for hypothetical small missing null or negative studies (Peters et al., 2007). As shown in Figure 4, seven missing studies were merged to obtain a symmetric funnel plot.

## Discussion

This study examined available learning burnout intervention studies, using meta-analyses to comprehensively evaluate the effects of the different interventions which were found to be overall effective. The interventions were broadly divided into individual, organizational, matching interventions, and learning engagement from a positive psychology perspective. Among these, the exercise intervention as an individual intervention, and group counseling—an organizational intervention—were the most common types of intervention.

Subgroup analysis of interventions showed that all interventions had significant effects, compared with the control group. Interestingly, the SMD of the meta-analysis on physical exercise was higher than that of group counseling. We speculate that the effects of the individual intervention (exercise intervention) were better than the ones of the organizational intervention (group counseling). Yet, group counseling was the most widely studied intervention, and all Chinese studies showed similar results. Group counseling improved students' self-efficacy in learning behavior and selecting positive coping strategies (Guo, 2016). A safe group atmosphere and a scientific goal process facilitate an interactive environment where students respect and understand each other, grow in their exploration, learning, and acceptance of themselves, reconstruct new attitudes and behaviors, and improve their interest in and satisfaction with life (Xu, 2019). In terms of physical activity, it may promote a good mood and is an important factor in psychological change. Negative emotions—depression and tension — generated during learning activities, decrease after exercise and psychological well-being increase significantly. Notably, the length and intensity, activity items, and physical status during exercise all affect psychological change (Lu et al., 2018). Interestingly, the studies that combined several interventions into an integrated intervention study, based on the factors of learning burnout, revealed the effectiveness of the integrated interventions (Tan, 2016; Wang et al., 2016). This aspect is noteworthy in the future.

Our analyses also showed that learning burnout intervention was effective among undergraduate, secondary vocational, and middle school students, compared to the control group. However, among postgraduates, junior college students, and schoolchildren there was an intervention effect but without statistical significance. The reason may be that people's psychological states vary over different life stages. During secondary and undergraduate stages students acquire intellectual skills that improve their learning of the intervention impact more effectively. Moreover, schoolchildren in the lower grades can initially control their emotional stress, but there are labile phenomena, most commonly experienced as anorexia. Once an anorexic tendency is seeded, a variety of poor learning habits and learning attitudes will ensue, followed by learning burnout (Liu, 2020). The effect formation, and embodiment of, learning burnout interventions is lasting and difficult to stabilize. Schoolchildren in higher grades are nearing puberty and become vulnerable to emotional and psychological problems (Duffield, 1998). The increased learning tasks and difficulty in learning may both affect the sense of achievement and satisfaction in learning. Students at the master's level have reached adulthood and mostly entered society. High educational level has a positive promoting effect on individuals' flexibility in coping problems and dispositions to use supporting psychological (Chen et al., 2014). Their psychology has stabilized, and general learning burnout interventions may have little effect on them psychologically. Another reason may be that there are few studies involving postgraduate, junior college students, and schoolchildren, and the combined results are biased toward reality, so more studies are needed to supplement the results in the future.

Subgroup analysis based on different scales showed that all interventions were effective. Maslach and Jackson (1981) compiled the Maslach Burnout Inventory (MBI) based on long-term observations and research among helping industry workers. Since then, most of the international scales on learning burnout have followed the design of this inventory. The OLBI-S developed by Demerouti et al. (2001) and based on the MBI, contains 16 items with two dimensions: exhaustion and disengagement (Reis et al., 2015).

Most of the scales used in China have been developed by Chinese scholars, based on the MBI, while considering the local context. The LBSCS, a three-dimensional model of depressed mood, inappropriate behavior, and low sense of achievement, has 35 items that measure the prevalence of burnout in college students' learning context (Lian et al., 2005). The ASBI has been applied to elementary and high school students and included three dimensions: physical and mental exhaustion, academic distancing, and low sense of achievement (Wu et al., 2010). Since it has only 16 items, it is widely adopted by Chinese scholars. The MSSLBS is a 21-item measure instrument with four dimensions, including emotional exhaustion, a sense of inefficient learning, teacher-student estrangement, and physical exhaustion (Hu and Dai, 2006).

The results of the subgroup analysis of intervention time showed unique results at different intervention times. Compared to the control group, the intervention group with an intervention period of 8 weeks and more, showed the best intervention effect for learning burnout. In some studies, the learning burnout interventions lasted 12, 16, or even 18 weeks (Wang, 2012; Sheng, 2016; Ezenwaji et al., 2019). Moreover, in other studies, follow-ups and remeasures after the learning burnout intervention were conducted to assess the delayed effect of the interventions. However, we did not conduct an analysis because few studies were involved. Also, their follow-up times varied, but their results showed a long-term intervention effect (Ni and Wu, 2009; Zhang et al., 2014; Guo, 2016; Wang, 2016; Ezenwaji et al., 2019; Shen, 2020). Interventions such as group counseling, exercise interventions, and time management training can have profound psychological effects on students and still be applied to their learning later in life.

Study heterogeneity was assessed using Cochran's χ^2^-based *Q*-test and the *I*^2^-test. Due to the large heterogeneity among the included studies, a random-effects model was adopted for the pooled analysis. We searched for possible sources of heterogeneity, using a meta-regression and a sensitivity analysis, but the results did not reveal any factors or studies that significantly affected the heterogeneity of the overall results. The funnel plot indicated significant publication bias of the studies, which had been corrected for the results using the trim-and-fill method, compensating for seven possible hypotheses of small missing zero or negative studies.

To the best of our knowledge, this is the first meta-analysis of learning-burnout intervention studies. We aimed to comprehensively evaluate the effect of different interventions on learning burnout. However, some limitations still affect the study results. First, considerable heterogeneity was observed in the analyses of the included studies. We tried several methods, including adopting a random effects model, and performed a meta-regression and a sensitivity analysis to identify factors or studies contributing to heterogeneity. Second, some subgroups contained few studies. Third, only Chinese and English literature were included in our study. And only one non-Asian randomized controlled trial was included. This may be due to the limitation of inclusion and exclusion criteria that excluded some studies with incomplete data or non-randomized controlled trials at the time of publication. It may also be due to the lack of relevant randomized controlled trials on the interventions of learning burnout in non-Asian. In addition, there may be equally relevant literature in other languages. For future studies, we recommend integrated interventions based on various factors of learning burnout and a focus on the long-term effects of the interventions on learning burnout. Furthermore, additional focus on interventions for learning burnout among primary school students is advised.

## Conclusion

This meta-analysis suggested that learning burnout interventions are effective. The subgroup analyses showed that group counseling was most widely used, exercise intervention was probably the most effective intervention, and 8 weeks or more was the appropriate intervention time. An integrated intervention study based on the factors of learning burnout is significant. More studies are needed to supplement the results in the future.

## Data Availability Statement

The original contributions presented in the study are included in the article/supplementary material, further inquiries can be directed to the corresponding author.

## Author Contributions

LT and FZ designed the study and created the first draft of the manuscript. LT performed the literature search, analyzed the data, and wrote the manuscript. LT, RY, and ZF article selection and quality appraisal. ZF suggested improvements. All authors contributed to the final manuscript and submission.

## Conflict of Interest

The authors declare that the research was conducted in the absence of any commercial or financial relationships that could be construed as a potential conflict of interest.

## References

[B1] Abarghouei M. R. Sorbi M. H. Abarghouei M. Bidaki R. Yazdanpoor S. (2016). A study of job stress and burnout and related factors in the hospital personnel of Iran. Electr. Phys. 8, 2625–2632. 10.19082/2625 27648189 PMC5014501

[B2] Al-Alawi M. Al-Sinawi H. Al-Qubtan A. Al-Lawati J. Habsi A. A. Al-Shuraiqi M.. (2017). Prevalence and determinants of burnout Syndrome and Depression among medical students at Sultan Qaboos University: a cross-sectional analytical study from Oman. Arch. Environ. Occup. Health 74, 1–10. 10.1080/19338244.2017.1400941 29116906

[B3] Bonini C. J. A. D. Sandra C. M. João M. (2011). Oldenburg burnout inventory - student version: cultural adaptation and validation into portuguese. Psicol. Reflexo Crítica 25, 709–718. 10.1590/S0102-7972201200040001029630412

[B4] Bresó E. Schaufeli W. B. Salanova M. (2010). Can a self-efficacy-based intervention decrease burnout, increase engagement, and enhance performance? a quasi-experimental study. Higher Educ. 61, 725–733. 10.1007/s10734-010-9334-6

[B5] Chen C. Huang J. Liang B. Y. University T. (2014). Psychological health diathesis assessment system: a nationwide survey of resilient trait scale for Chinese adults. Psychol. Behav. Stud. 12, 735–742. 10.3969/j.issn.1672-0628.2014.06.004

[B6] Chen S. S. Zhang R. S. Chen J. (2012). Effect of group intervention on college students' learning burnout. Psychol. Behav. Stud. 10, 138–142. 10.3969/j.issn.1672-0628.2012.02.010

[B7] Chen Y. J. (2020). Study on the Relationship and Intervention Between Core Self-Evaluation, School Sense of Belonging and Learning Burnout of Secondary Vocational Students. Hebei: Hebei University.

[B8] Chou L. P. Li C. Y. Hu S. C. (2014). Job stress and burnout in hospital employees: comparisons of different medical professions in a regional hospital in Taiwan. BMJ Open. 4:e004185. 10.1136/bmjopen-2013-004185 24568961 PMC3939670

[B9] Cumpston M. Li T. Page M. J. Chandler J. Thomas J. (2019). Updated guidance for trusted systematic reviews: a new edition of the Cochrane handbook for systematic reviews of interventions. Cochrane Database Syst. Rev. 10:ED000142. 10.1002/14651858.ED000142 31643080 PMC10284251

[B10] Dai Y. F. (2014). The Effect of Training in Learning Engagement on the Academic Achievement and Learning Burnout. Zhejiang: Zhejiang Normal University.

[B11] Demerouti E. Bakker A. B. Nachreiner F. Schaufeli W. B. (2001). The job demands-resources model of burnout. J. Appl. Psychol. 86, 499–512. 10.1037/0021-9010.86.3.499 11419809

[B12] Duffield C. M. (1998). Adolescent health: the role of individual differences. Accident Emerg. Nurs. 6:176. 10.1016/S0965-2302(98)90041-8

[B13] Dyrbye L. N. Thomas M. R. Massie F. S. Power D. V. Eacker A. Harper W.. (2008). Burnout and suicidal ideation among U.S. medical students. Ann. Internal Med. 149, 334–341. 10.7326/0003-4819-149-5-200809020-00008 18765703

[B14] Egger M. Smith G. D. Schneider M. Minder C. (1997). Bias in meta-analysis detected by a simple, graphical test. BMJ 315, 629–634. 10.1136/bmj.315.7109.629 9310563 PMC2127453

[B15] Elloy D. F. Terpening W. Kohls J. (2001). A causal model of burnout among self-managed work team members. J. Psychol. 135, 321–334. 10.1080/00223980109603702 11577974

[B16] Ezenwaji I. O. Eseadi C. Ugwoke S. C. Vita-Agundu U. C. Agu M. A. (2019). A group-focused rational emotive behavior coaching for management of academic burnout among undergraduate students: implications for school administrators. Medicine 98:e16352. 10.1097/MD.0000000000016352 31348235 PMC6708802

[B17] Freudenberger H. J. (1974). Staff burnout. J. Soc. Issues 30, 159–165. 10.1111/j.1540-4560.1974.tb00706.x

[B18] Guo X. Q. (2016). The Influencing Factors of College Students' Learning Burnout and Its Intervention Study. Shanxi: Shanxi Medical University.

[B19] Higgins J. P. Thompson S. G. (2002). Quantifying heterogeneity in a meta-analysis. Stat. Med. 21, 1539–1558. 10.1002/sim.1186 12111919

[B20] Hu Q. Dai C. L. (2006). A research on middle school students' learning burnout structure. J. Psychol. Sci. 1, 162–164. 10.3969/j.issn.1671-6981.2007.01.041

[B21] Huang D. X. (2018). Study on the Relationship and Intervention Between Positive Emotion and Learning Burnout High School Students. Liaoning: Shenyang Normal University.

[B22] Jackson E. R. Shanafelt T. D. Hasan O. Satele D. V. Dyrbye L. N. (2016). Burnout and alcohol abuse/dependence among U.S. medical students. Acad. Med. 91, 1251–1256. 10.1097/ACM.0000000000001138 26934693

[B23] Jadad A. R. Moore R. A. Carroll D. Jenkinson C. Reynolds D. J. M. Gavaghan D. J.. (1996). Assessing the quality of reports of randomized clinical trials: is blinding necessary? Control. Clin. Trials 17, 1–12. 10.1016/0197-2456(95)00134-4 8721797

[B24] Khalaj E. Savoji A. P. (2018). The effectiveness of cognitive self-regulatory education on academic burnout and cognitive dissonance and academic achievement of elementary students. World Fam. Med. 16, 225–231. 10.5742/MEWFM.2018.93224

[B25] Kurzman P. A. (1981). Book review: burnout: from tedium to personal growth. Soc. Casework 62, 622–623. 10.1177/104438948106201007

[B26] Li J. (2016). Study on the Effect of Affective Teaching on Learning Burnout of High School Students. Jiangsu: Nanjing Normal University.

[B27] Li Y. Y. (2019). The Effect of Group Counseling on Junior Middle School Students' Academic Achievements at School. Zhejiang: Zhejiang Normal University.

[B28] Lian R. Yang L. X. Wu L. H. (2005). Relationship between professional commitment and learning burnout of undergraduates and scales develop. Acta Psychol. Sin. 5, 632–636.

[B29] Liu G. H. (2020). Psychological analysis and countermeasure research of primary school students' weariness of learning. Modern Commun. 18, 145–147.

[B30] Liu Y. J. (2013). Effect of comprehensive intervention on learning burnout of college students. China J. Health Psychol. 21, 1843–1845. 10.13342/j.cnki.cjhp.2013.12.017

[B31] Lu Y. R. Wang Y. Chen Y. H. (2018). Research on Sports Culture and Health Education. Beijing: Xinhua Publishing House.

[B32] Luo X. (2012). A Study on the Relationship Between Learning Burnout, Attributional Style and Personality Factor of Master Graduate. Liaoning: Liaoning Normal University.

[B33] Luo Z. Y. (2012). The Effect of Time Management Training on Achievement Motivation and Learning Burnout of Middle School Students. Shanxi: Shanxi Medical University.

[B34] Maslach C. Jackson S. E. (1981). The measurement of experienced burnout. J. Organ. Behav. 2, 99–113. 10.1002/job.4030020205

[B35] Maslach C. Leiter M. P. (1997). The Truth about Burnout: How Organizations Cause Personal Stress and What to do about It. San Francisco, CA: Jossey-Bass.

[B36] May R. W. Bauer K. N. Fincham F. D. (2015a). School burnout: diminished academic and cognitive performance. Learn. Individual Differ. 42, 126–131. 10.1016/j.lindif.2015.07.015

[B37] May R. W. Sanchez-Gonzalez M. A. Fincham F. D. (2015b). School burnout: increased sympathetic vasomotor tone and attenuated ambulatory diurnal blood pressure variability in young adult women. Stress 18, 11–19. 10.3109/10253890.2014.969703 25256608

[B38] Meier S. F. Schmeck R. R. (1985). The burned-out college student: a descriptive profile. J. College Student 1, 63–69.

[B39] Moher D. Liberati A. Tetzlaff J. Altman D. G. (2009). Preferred reporting items for systematic reviews and meta-analyses: the PRISMA statement. PLOS Med. 6:e1000097. 10.1371/journal.pmed.1000097 19621072 PMC2707599

[B40] Neumann Y. Finaly-Neumann E. Reichel A. (1990). Determinants and consequences of students' burnout in universities. J. Higher Educ. 61, 20–31. 10.2307/1982032

[B41] Ni S. G. Wu X. C. (2009). Study on the effect of cognitive behavior interactive group counseling on college students' academic burnout. Chin. J. Clin. Psychol. 17, 512–514.

[B42] Overton R. C. (1998). A comparison of fixed-effects and mixed (random-effects) models for meta-analysis tests of moderator variable effects. Psychol. Methods 3, 354–379. 10.1037/1082-989X.3.3.354

[B43] Peters J. Sutton A. Jones D. Abrams K. Rushton L. (2007). Performance of the trim and fill method in the presence of publication bias and between-study heterogeneity. Stat. Med. 26, 4544–4562. 10.1002/sim.2889 17476644

[B44] Pines A. M. (2000). Treating career burnout: a psychodynamic existential perspective. J. Clin. Psychol. 56, 633–642. 10.1002/(SICI)1097-4679(200005)56:5<633::AID-JCLP5>3.0.CO;2-# 10852150

[B45] Reis D. Xanthopoulou D. Tsaousis I. (2015). Measuring job and academic burnout with the Oldenburg Burnout Inventory (OLBI): factorial invariance across samples and countries. Burnout Res. 2, 8–18. 10.1016/j.burn.2014.11.001

[B46] Santos B. R. A. D. Eduardo P. C. Antonio D. O. M. Giancarlo L. Fregnani José H. T. G. Ribeiro P. B. S. (2018). Burnout among medical students during the first years of undergraduate school: prevalence and associated factors. PLoS ONE 13:e0191746. 10.1371/journal.pone.0191746 29513668 PMC5841647

[B47] Schaufeli W. B. Martinez I. M. Pinto A. M. Salanova M. Bakker A. B. (2002). Burnout and engagement in university students. J. Cross Cult. Psychol. 33, 464–481. 10.1177/0022022102033005003

[B48] Shang D. M. (2016). The application of focus-oriented group counseling in learning burnout intervention of higher vocational students. Shanxi Youth 5, 22–23.

[B49] Shen D. (2020). Study on the design of group counseling program and the effect of intervention on college students' learning burnout. China J. Health Psychol. 28, 453–457. 10.13342/j.cnki.cjhp.2020.03.030

[B50] Sheng W. (2016). Sports Intervention Experimental Study of the Impact of High School Girls Learning Burnout. Jiangsu: Suzhou University.

[B51] Shi Y. (2017). Study on the Influencing Factors and Intervention of Junior Middle School Students' Learning Burnout. Shanghai: Shanghai Normal University.

[B52] Skodova Z. Lajciakova P. (2013). The effect of personality traits and psychosocial training on burnout syndrome among healthcare students. Nurse Educ. Today 33, 1311–1315. 10.1016/j.nedt.2013.02.023 23545453

[B53] Slivar B. (2001). The syndrome of burnout, self-image, and anxiety with grammar school students. Psihološka Obzorja 10, 21–32.

[B54] Tan C. H. (2016). A Research on the Students' Current Lassitude Condition and Intervention in grade Two of Junior High School. Hebei: Hebei University.

[B55] Tavolacci M. P. Veber B. (2015). Burnout and stress in medical students in France: prevalence and associated factors. Euro. J. Public Health 25, 161–162. 10.1093/eurpub/ckv171.084

[B56] Wan Y. (2020). The effect of mindfulness group intervention on college students' mental health. Psychologies 15:28. 10.19738/j.cnki.psy.2020.13.01833493652

[B57] Wang C. Q. Bao L. J. Cui L. Z. (2016). The effect of psychological health exercise combined with group counseling on relieving middle school students' learning burnout. Chin. J. School Health 37, 773–775. 10.16835/j.cnki.1000-9817.2016.04.024

[B58] Wang D. Mou Z. Y. Zhai J. X. Zong H. X. Zhao X. D. (2008). Study on Stata software in investigating publication bias in meta-analysis. Modern Prev. Med. 15, 2819–2822. 10.3969/j.issn.1003-8507.2008.15.002

[B59] Wang L. H. (2016). Intervention effect of time management group training on learning burnout of secondary vocational students. Chin. J. School Health 37, 557–560. 10.16835/j.cnki.1000-9817.2016.05.043

[B60] Wang M. (2012). The effect of physical education teaching methods on college students' learning burnout. Sport 11, 61–62. 10.3969/j.issn.1674-151X.2012.11.033

[B61] Wu X. Q. (2017). The Investigation and Intervention Study on Learning Burnout of Higher Vocational Students. Zhejiang: Zhejiang Normal University.

[B62] Wu Y. Dai X. Y. Wen Z. L. (2010). The Development of Adolescent Student Burnout Inventory. Chin. J. Clin. Psychol. 18, 152–154. 10.16128/j.cnki.1005-3611.2010.02.018

[B63] Wu Y. P. (2017). The Influence of Imaginary Contact on Learning Burnout of Secondary Vocational Students. Fujian: Fujian Normal University.

[B64] Wu Y. Y. (2018). The Timely Review's Impact of High School Students on Chemical Academic Performance and Burnout. Zhejiang: Zhejiang Normal University.

[B65] Xi L. L. (2019). Study on the relationship and intervention between learning burnout and professional commitment of nursing students of college. Health Vocational Educ. 37, 115–117.

[B66] Xia Q. Yang S. Q. (2014). An experimental study on improving learning burnout of primary school students by mental health education. J. North China Univ. Sci. Technol. 2, 205–207. 10.19539/j.cnki.2095-2694.2014.02.038

[B67] Xiao X. (2013). The Structural Characteristics and Psychological Intervention on Learning Burnout of Graduate Students. Hubei: Wuhan Sports Institute.

[B68] Xiong X. M. Fang X. P. (2017). Research on the academic burnout of independent college students in Jiangxi province and its intervention. J. Jiangxi Sci. Technol. Normal Univ. 2, 83–91. 10.3969/j.issn.1007-3558.2017.02.013

[B69] Xu L. L. (2019). Intervention study of group counseling in orientation of professional quality on college students' academic burnout. J. Beijing Univ. Chem. Technol. 4, 97–102. 10.3969/j.issn.1671-6639.2019.04.016

[B70] Xu Z. H. (2018). A Study on the Relationship and Intervention Between Core Self-Evaluation, Academic Social Comparison and Learning Burnout in Senior High School Students. Shanghai: Shanghai Normal University.

[B71] Yang C. M. (2014). An Experimental Study on the Influence of Enterprise-Oriented Borderless Class Management on Learning Burnout and Personal Growth Initiative of Secondary Vocational Students. Yunnan: Yunnan Normal University.

[B72] Yao J. J. (2015). The Applied Research of the Learning Burnout in Left-behind Junior High School Students. Hebei: Hebei Normal University.

[B73] Ye X. Lin X. H. Zhou Y. Y. (2017). The effect of group mindfulness practice on the learning burnout of college students. J. Fujian Med. Univ. 18, 37–40.

[B74] Zhang P. Tang Q. Du Y. C. Fu L. Zheng T. (2014). The effect of group counseling on leaning burnout students in engineering college. J. Sichuan Normal Univ. 37, 287–292. 10.3969/j.issn.1001-8395.2014.02.026

[B75] Zhang R. (2017). A Study of Related Factors of Mobile Phone Dependence in High School Students and the Group Psychological intervention. Shanxi: Shanxi Medical University.

[B76] Zhang S. Y. (2018). Study on the Status and Intervention on Learning Burnout of Secondary Vocational Students. Jiangsu: Yangzhou University.

[B77] Zhang T. (2015). Mindfulness-Based Stress Reduction for High School Students' Academic Burnout. Gansu: Northwest Normal University.

[B78] Zhao R. F. (2019). The Relationship between Resilience and Learning Burnout for High-Level Graders in Primary School and Intervention Study. Hebei: Hebei Normal University.

[B79] Zhou H. (2019). Study on the Influence and Intervention of Mindfulness on Learning Burnout of Art Students. Jiangxi: Jiangxi Normal University.

[B80] Zhou X. H. (2011). Experimental research on physical exercises and college students' academic burnout and self-harmony. China J. Health Psychol. 19, 461–463. 10.13342/j.cnki.cjhp.2011.04.018

[B81] Zhu D. D. (2016). Study on the Status of Learning Burnout of Junior Middle School Students and the Box Room Intervention. Jiangsu: Yangzhou university.

